# Evaluation of ammonia fibre expansion (AFEX) pretreatment for enzymatic hydrolysis of switchgrass harvested in different seasons and locations

**DOI:** 10.1186/1754-6834-3-1

**Published:** 2010-01-04

**Authors:** Bryan Bals, Chad Rogers, Mingjie Jin, Venkatesh Balan, Bruce Dale

**Affiliations:** 1 Biomass Conversion Research Laboratory, Department of Chemical Engineering and Material Science, Michigan State University, Lansing, MI, USA

## Abstract

**Background:**

When producing biofuels from dedicated feedstock, agronomic factors such as harvest time and location can impact the downstream production. Thus, this paper studies the effectiveness of ammonia fibre expansion (AFEX) pretreatment on two harvest times (July and October) and ecotypes/locations (Cave-in-Rock (CIR) harvested in Michigan and Alamo harvested in Alabama) for switchgrass (*Panicum virgatum*).

**Results:**

Both harvest date and ecotype/location determine the pretreatment conditions that produce maximum sugar yields. There was a high degree of correlation between glucose and xylose released regardless of the harvest, pretreatment conditions, or enzyme formulation. Enzyme formulation that produced maximum sugar yields was the same across all harvests except for the CIR October harvest. The least mature sample, the July harvest of CIR switchgrass, released the most sugars (520 g/kg biomass) during enzymatic hydrolysis while requiring the least severe pretreatment conditions. In contrast, the most mature harvest released the least amount of sugars (410 g/kg biomass). All hydrolysates were highly fermentable, although xylose utilisation in the July CIR hydrolysate was poor.

**Conclusions:**

Each harvest type and location responded differently to AFEX pretreatment, although all harvests successfully produced fermentable sugars. Thus, it is necessary to consider an integrated approach between agricultural production and biochemical processing in order to insure optimal productivity.

## Background

The conversion of lignocellulosic biomass to ethanol is a renewable, environmentally friendly alternative to oil for transportation fuel. Although agricultural residues such as corn stover and wheat straw will most likely play a significant role, dedicated energy crops must also be grown in order to make enough biofuels. Gallagher *et al*., for example, estimate 7.9 billion gallons of ethanol per year can be produced from corn stover (assuming 80 gallons per dry ton) [1]. However, the US Energy Security and Investment Act of 2007 mandates 16 billion gallons of cellulosic ethanol by 2022.

Switchgrass (*Panicum virgatum*) is a native North American perennial grass, commonly cited as a potential dedicated bioenergy feedstock. Intensive research on switchgrass production has been successful in increasing yields [2], and a recent on-farm study suggests that it can be produced for as little as US$46 per metric tonne [3]. The Oak Ridge National Laboratory predicts that 171 million tons of switchgrass can be produced economically within the US [4].

Despite the potential for ethanol from switchgrass, several questions remain regarding best production and harvesting practices. Several cultivars have been developed from both switchgrass ecotypes, upland and lowland. In general, lowland cultivars provide the highest yields at lower latitudes, but upland varieties are more suited for a cooler climate [5]. In addition, several papers have proposed multiple harvests per year. Fike *et al*. reported 36% increase in biomass yield for upland and 8% increase for lowland cultivars of switchgrass when harvesting in both June and October compared to one harvest in October [6]. Monti *et al*. observed similar trends for the first 2 years of harvests, yet in later years the multiple-harvest scenario had lower yields than a single harvest [7]. Despite the possibility of lower yields, there may be other advantages to performing multiple harvests. An earlier harvest will likely contain more digestible carbohydrates, thus potentially leading to greater sugar recovery. In addition, immature switchgrass may provide other valuable coproducts such as protein that could improve the overall economics of a biorefinery [8,9].

As with production and harvesting, several options are available for pretreatment and conversion of switchgrass to ethanol [10]. In general, switchgrass responds favourably to pretreatment and hydrolysis, with several studies reporting greater than 90% conversion of cell wall carbohydrates to sugars [11,12]. These studies vary considerably in the type of pretreatment used as well as hydrolysis and fermentation conditions, and thus are not directly comparable. In addition, some studies did not specify the type and harvest date of the switchgrass used, thus making it difficult to assess these results. Dien *et al*. observed declining sugar yields in late maturity switchgrass relative to earlier maturities when subjected to dilute acid pretreatment and enzymatic hydrolysis [13]. To date, we do not know of any study that compares the response of different cultivars or harvest locations of switchgrass to pretreatment and hydrolysis.

Ammonia fibre expansion (AFEX) is a promising method for pretreating agricultural material for bioenergy production. During this process, liquid ammonia is added to the biomass under moderate pressure (100 to 400 psi) and temperature (70 to 200°C) before rapidly releasing the pressure. Major process parameters are the temperature of the reaction, residence time, ammonia loading, and water loading. This process decrystallises the cellulose, hydrolyses hemicellulose, removes and depolymerises lignin, and increases the size and number of micropores in the cell wall, thereby significantly increasing the rate of enzymatic hydrolysis [14]. In previous studies, AFEX treatment resulted in near theoretical yields of glucose on different types of agricultural residues [15] and energy crops [16,17]. In particular, previous work has shown conversions of over 90% of the glucan and 70% xylose on switchgrass, although again the cultivar/ecotype and harvest date was not specified [18].

Thus, the goal of this paper is to determine the effectiveness of AFEX pretreatment on two harvest times (July and October) and ecotypes/locations (upland switchgrass in Michigan and lowland switchgrass in Alabama) for switchgrass. In particular, the response of each switchgrass sample to variations in the four pretreatment parameters, different enzyme loadings, and fermentation is considered for this work. These responses are compared across harvest dates and locations in order to assess the potential for multiple harvest systems and the variability in switchgrass yields, respectively.

## Methods

### Feedstock

Two varieties of switchgrass were used for this study. Alamo (a lowland variety) switchgrass, grown at Auburn University (Auburn, AL, USA), was harvested in mid July and mid October 2005. Cave-in-Rock (CIR) switchgrass (an upland variety) was grown at Michigan State University (East Lansing, MI, USA) and harvested in early July and mid October 2008. All varieties were milled using a Fitzpatrick JT-6 Homoloid hammer mill (Continental Process Systems, Westmont, IL, USA) to a mesh size of 2 mm. Samples were dried to less than 10% moisture and stored at 2°C until use.

### AFEX conditions experiment

For screening AFEX conditions, pretreatment was performed in a 22 ml reactor. Switchgrass was premixed with water at the desired loading and 3 g dry weight was added to the reactor before being sealed shut. Air was removed from the reactor using a vacuum. Anhydrous ammonia was preheated to a desired pressure in the ammonia loading vessel, and the biomass preheated to the desired temperature. Both the ammonia pressure and biomass temperature were chosen in order to reach a specified temperature once the ammonia was added to the biomass. The heat of mixing between ammonia and water raises the temperature beyond the preheated values, and a precise final temperature is therefore difficult to obtain. Instead, a range of preheated values was used to obtain a range of temperatures, and the final temperature of the biomass was recorded and used. Pressure was released at the end of the desired residence time by turning a ball valve. After the reaction, the biomass was removed and allowed to dry in a fume hood overnight. Based on previous studies [15], it was assumed that no net change in the biomass weight occurred during pretreatment.

AFEX conditions ranged from 0.4 to 2 g ammonia/g dry biomass, 0.4 to 2 g water/g dry biomass, and 5 to 30 min residence time. In addition, the temperature range was generally between 80 to 200°C. At least 45 total AFEX conditions were chosen for each type of switchgrass tested. The 'corner points' of ammonia and water were specifically chosen at a moderate temperature and pressure. In addition, a near centre point (1 g ammonia/g dry biomass, 1 g water/g dry biomass, 15 min residence time, moderate temperature) was replicated multiple times. The remaining conditions were assigned randomly.

Enzymatic hydrolysis was performed at 3% dry biomass loading and 15 ml total volume. A 0.05 M citrate buffer was used to keep the pH constant at approximately 5.0. Tetracycline and cycloheximide were added to prevent microbial and fungal growth. Accelerase (Genencor, Rochester, NY, batch no. 1600844643) was used as the cellulase and loaded at 5 mg Accelerase/g dry biomass (3.2 filter paper units (FPU)/g dry biomass). Hydrolysates were incubated at 50°C and rotated at 200 rpm for 72 h. After the incubation period, enzymes were deactivated by heating samples to 99°C. Monomeric glucose and xylose concentration was determined through high performance liquid chromatography (HPLC) using a BioRad (Richmond, CA, USA) Aminex HPX-87P carbohydrate analysis column. Degassed HPLC water with a flow rate of 0.6 ml/min was used as the mobile phase, while the temperature in the column was kept constant at 85°C.

Sugars are reported as the total monomeric glucose and xylose released from the biomass during enzyme hydrolysis. This includes sugars produced from hydrolysis of polymeric sugars as well as soluble sugars that were not degraded during pretreatment. No attempt was made to determine the degradation of soluble sugars during pretreatment or the conversion of polysaccharides to monomeric sugars.

A reduced linear model based on the total monomeric glucose and xylose released during enzymatic hydrolysis was used to analyse the results using Minitab 15 (Minitab, State College, PA, USA) as the statistical software package. Ammonia loading, water loading, residence time, and the final reaction temperature were used as parameters as well as all interaction effects. Any parameter or interaction term that did not have a significant effect (*P *< 0.05) was eliminated. The final model was used to analyse the response of total sugar yield to each pretreatment parameter as well as to estimate the optimal AFEX conditions for each switchgrass sample.

### Enzyme addition experiment

For all subsequent experiments described in this paper, AFEX was performed at the estimated optimal conditions determined from the above experiments. The same treatment method was used except that AFEX was performed in a 1.5 l reactor rather than a 22 ml reactor. Between 80 to 150 g dry switchgrass was used for each batch. The amount of switchgrass depended on the ammonia loading, as a practical limitation of the ammonia loading vessel was 160 g. Multiple batches of AFEX treatment were performed, and no significant differences (*P *< 0.05) were seen in sugar released through enzymatic hydrolysis between batches. All batches were then combined before proceeding with further experiments.

Four commercial enzymatic mixtures were used in these experiments: Accelerase, the b-glucosidase Novozyme 188 (Novozymes, Cambridge, MA, batch no. 058K1144), Multifect Xylanase (Genencor, batch no. 4900805391), and Multifect Pectinase (Genencor, batch no. 4010833580). Enzyme concentrations were determined by nitrogen analysis using a Skalar Primacs SN Total Nitrogen Analyser (Breda, The Netherlands), which uses the Dumas method of combusting all nitrogen to NOx. Enzyme loading varied between 5 to 20 mg/g biomass for Accelerase and 0 to 10 mg/g biomass for the other enzyme mixtures. A total of 48 hydrolysis experiments were run for each type of switchgrass, representing 25 different enzyme combinations determined using the Box-Behnken method [19]. Hydrolysis was performed in the manner stated above. Results were analysed with Minitab 15 using response surface methodology to determine the importance of each type of enzyme in releasing sugars.

### Rate determination

For the rate experiments, the estimated optimal enzyme loading for each biomass was used as determined from the previous experiment. Hydrolysis was performed in 250 ml flasks with a working volume of 100 ml. All other conditions were as stated previously. Samples of 1 ml were taken at 0, 3, 6, 10, 24, 72, and 168 h. Cut pipette tips were used in order to sample both solids and liquid from the flasks, thereby preventing bias in later hydrolysis time periods due to changing the solid loading.

### Fermentation

Fermentation studies were carried out using *Saccharomyces cerevisiae *424A (LNH-ST), a yeast strain genetically modified at Purdue University, West Lafayette, IN, USA, to coferment xylose into ethanol. The yeast was grown on yeast extract phosphate (YEP) media before transferring to switchgrass hydrolysate at an initial cell density of 2.0 OD at 600 nm (approximately 0.95 g/l). The hydrolysates used were at 20% solid loading, with the exception of the October Alamo harvest which was grown at 10% solid loading due to lack of available sample material. All hydrolysates were prepared as mentioned above with the exception that 50 mg/l chloramphenicol was used as the antibiotic. Because a 20% solid loading mixture has no standing water and is thus unable to be mixed properly in a shake flask, the biomass and enzyme was added in a fed-batch manner to allow the fibre structure to break down. It was found that adding half of the enzyme and biomass immediately and half after 3 h residence time was sufficient to insure flowability. After hydrolysis, the solids and liquids were separated and fermentation was performed on the liquid portion only. Duplicate hydrolysates were combined together prior to fermentation, and fermentation was carried out in duplicates. No detoxification of the hydrolysate or nutrient supplementation of the fermentation media was performed [20]. Fermentation was carried out at 30°C, 150 rpm in unbaffled Erlenmeyer flasks.

### Composition analysis

Switchgrass cell wall composition was determined based upon the standard method described by the National Renewable Energy Laboratory (NREL) [21]. Total extractives were determined using an accelerated solvent extractor with water followed by ethanol as the solvent at 1, 500 psi. A portion of the water extract was analysed via HPLC for soluble sugars. For total carbohydrate analysis, extracted switchgrass was hydrolysed in 72% sulfuric acid at 30°C for 1 h, followed by 1 h hydrolysis in 4% sulfuric acid at 121°C. The resulting hydrolysate was filtered, and the remaining solids were gravimetrically analysed to determine acid-insoluble lignin. Total sugars released within the hydrolysate were analysed using a BioRad Aminex 87 H column with a constant flow rate of 0.6 ml/min using 5 mM sulfuric acid and a temperature of 65°C. Ash content was gravimetrically determined by combusting at 575°C for 16 h. All composition experiments were performed in triplicate. Significant differences between harvests were determined using a single-factor analysis of variance at a 95% confidence interval.

## Results and Discussion

### Compositional analysis

Table 1 shows the composition of the four harvests of switchgrass. There were significant differences in glucan and xylan content in CIR switchgrass, but not Alamo switchgrass. Of particular importance is the lignin, which increases from 10.4% to 16.7% between the July and October harvest of CIR switchgrass. The amount of lignin present has been linked to poor hydrolysis yields, particularly as AFEX does not remove lignin. However, increased glucan and xylan content shows the potential for the later harvest to achieve higher overall sugar yields. Both xylan and lignin showed a general trend of increasing with increasing maturity, as the July harvest of the northern CIR switchgrass contained the least amount of both compounds between all harvests, while the October harvest of CIR contained the most xylan, and the two October harvests both contained high lignin. The CIR switchgrass, grown in Michigan, experienced a shorter growing season than the Alamo harvests, and thus the July harvest of CIR is less mature than Alamo, while the October harvest of CIR is more mature than Alamo, as the northern variety tends to senesce earlier in the year.

**Table 1:** Composition analysis (as percentage of total dry weight) for the July and October harvests of Cave-in-Rock (CIR) and Alamo switchgrass.

	CIR: July	CIR: October	Alamo: July	Alamo: October
Glucan^a^	30.6 ± 0.2^d^	33.6 ± 0.5^e^	32.6 ± 0.3^f^	32.9 ± 0.2^f^
Sucrose	5.1 ± 0.3^d^	2.4 ± 0.2^e^	3.8 ± 0.2^f^	3.8 ± 0.4^f^
Xylan	19.4 ± 0.3^d^	25.3 ± 0.2^e^	22.8 ± 0.1^f^	23.0 ± 0.2^f^
Arabinan	2.0 ± 0.1^d^	2.0 ± 0.1^f^	1.8 ± 0.1^e^	2.0 ± 0.1^d^
Lignin^b^	10.4 ± 0.4^d^	16.7 ± 0.5^e^	15.4 ± 0.4^f^	17.2 ± 0.2^e^
Solubles^c^	26.0 ± 0.6^d^	15.8 ± 0.1^e^	18.1 ± 0.2^f^	15.0 ± 0.2^g^
Ash	5.0 ± 0.06^d^	3.9 ± 0.02^e^	2.5 ± 0.03^f^	2.0 ± 0.01^g^
Closure	95.4 ± 0.8	97.3 ± 0.7	93.2 ± 0.6	92.1 ± 0.4

Different letters (d, e,f,g) represent significant differences (*P *< 0.05) in composition across the different feedstocks.

^a^Does not include soluble glucose; ^b^acid insoluble (Klason) lignin; ^c^includes both water and ethanol solubles.

As expected, solubles and ash decreased for later harvests, as the grass senesces at the end of the growing season and mobilises those compounds for storage in the root system. CIR switchgrass also tended to contain more solubles and ash than the Alamo switchgrass. Overall mass closure ranged from 92% to 97%. Remaining material may include acid-soluble lignin, galactan and other minor sugars, and insoluble protein. Acid soluble lignin in particular can range from 2% to 6% of the total composition [22,23]. AFEX does not remove any material from the biomass, and so this acid soluble lignin may still impact hydrolysis yields.

### AFEX conditions

**Figure 1:**
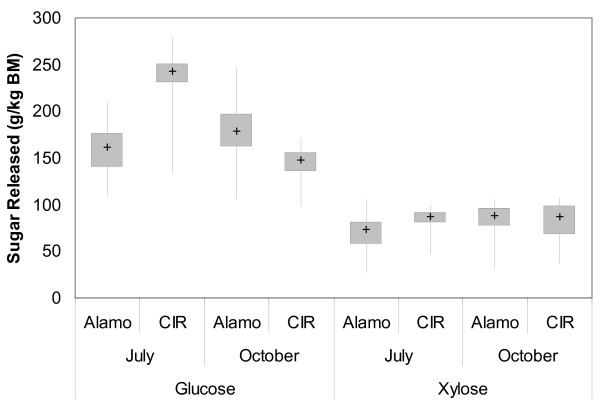
**Box and whisker plot for sugar yields at varying pretreatment conditions**. Minimum and maximum values, as well as the interquartile range, are shown. Median values are represented by the + sign. Pretreatment conditions were varied as stated in the text. For all data points, hydrolysis was performed at 3% solid loading, 50°C, 200 rpm rotation, and 5 mg Accelerase/g dry biomass, with samples collected after 72 h.

The range of glucose and xylose yields is shown in Figure 1. In general, the release of glucose and xylose was similar across AFEX conditions; that is, AFEX conditions that produced high glucose yields also produced high xylose yields. This correlation was higher for the October harvests for both plantings of switchgrass than the July harvests. Xylose yields are within the same range for all harvests of switchgrass, with most samples between 50 to 100 g xylose/kg switchgrass depending upon the conditions used. Maximum xylose yields were similar across all harvests. The amount of glucose released tended to be higher for CIR switchgrass harvested in July relative to all other harvests. In contrast, the October harvest of CIR switchgrass tended to have the lowest glucose yields, with a maximum glucose yield of 172 g/kg switchgrass.

Furthermore, different AFEX conditions did not greatly affect glucose yields for the October harvest of CIR switchgrass, with an inner quartile range of only 135 to 156 g/kg switchgrass. In comparison, Alamo October harvest had a broader range, with an inner quartile range of 162 to 197 g/kg switchgrass and a maximum yield of 247 g/kg. The July harvest of Alamo switchgrass had similar yields for its inner quartile range, but the maximum sugar yield (209 g/kg) was well below the October harvest. Thus, while most pretreatment conditions show a similar digestibility for both harvests of Alamo switchgrass, the best AFEX conditions tended to produce greater glucose release for the October harvest.

For the CIR switchgrass, the October harvest required more severe pretreatment conditions (higher temperature, more ammonia, and/or longer residence times) than the July harvest, as seen in Table 2. In contrast, both harvests of Alamo switchgrass had similar pretreatment conditions, although the October harvest required a greater ammonia loading. In all cases, the models gave a reasonable approximation of the results, with R^2 ^values at or above 80%. AFEX is a complex physiochemical pretreatment, resulting in multiple changes to the cell wall structure. One primary effect is to solubilise and remove lignin and hemicellulose from the cellulose. Because the CIR July switchgrass has less lignin and hemicellulose than the other harvests, it may require less extreme conditions to properly solubilise and remove this material.

**Table 2:** Optimal ammonia fibre expansion (AFEX) conditions within the parameters tested in this study.

	CIR: July	CIR: October	Alamo: July	Alamo: October
Ammonia (g/g BM)	0.9	2.0	1.6	2.0
Water (g/g BM)	0.4	0.4	2.0	2.0
Residence time (min)	20	30	30	25
Temperature (°C)	80	130	160	150
Sugar yield (g/kg BM)	385	280	288	320
Sugar yield (%)	65.6	41.6	45.0	49.5
Glucose yield (g/kg BM)	276	172	198	221
Xylose yield (g/kg BM)	98	102	89	100
R^2^	80.0%	79.9%	83.6%	94.1%

BM = biomass; CIR = Cave-in-Rock.

Although most pretreatment conditions affected the glucose and xylose yields in a similar manner, xylose yields tended to be lower than expected at high temperatures. Currently unpublished research in our laboratory suggests that xylan is degraded at high temperatures. Likewise, soluble sugars also degrade at high temperatures. These issues may partially explain why both CIR harvests require low temperatures relative to the Alamo harvests. The CIR July switchgrass is high in sucrose, and thus requires low temperatures to preserve these sugars, whereas the CIR October harvest had highly indigestible cellulose. Because the glucose released was low and did not greatly vary across all pretreatment conditions, a lower temperature was required to obtain high overall sugar release.

The differences in water requirements for the two types of switchgrass used is pronounced. Alamo switchgrass required high water to biomass ratios while CIR switchgrass required low water to biomass ratio. Water has a complex role in AFEX pretreatment, as it acts as a competing catalyst for breaking acetyl bonds, moderates the pH, and aids in solubilising ammonia. In addition, high ammonia concentrations (relative to water) act to convert crystalline cellulose to the more digestible cellulose III [24]. This may be the reason for the high ammonia/low water requirement for CIR October switchgrass. If so, a more anhydrous AFEX pretreatment may improve glucose yields for this harvest. For the CIR July harvest, the water loading is highly dependent upon the temperature of the process. At high temperatures, high water loadings must be used, while low loadings are required for high yields at low temperatures. The high water loadings at high temperatures may act as a moderating influence on sugar degradation by ammonia reactions, as it reduces the ammonia concentration and pH of the reaction.

### Enzyme conditions

**Figure 2:**
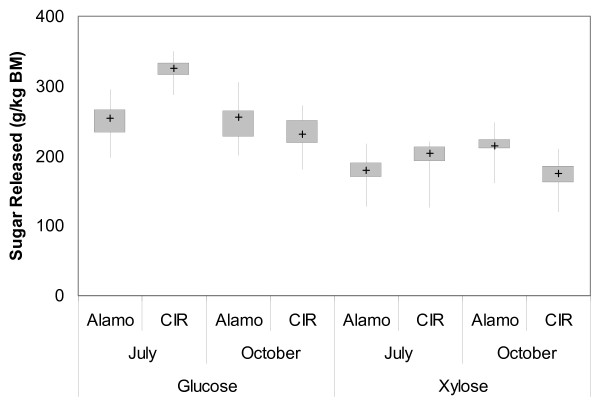
**Box and whisker plot for sugar yields at varying enzyme loadings**. Ammonia fibre expansion (AFEX) pretreatment was kept constant for each harvest (conditions listed in Table 2), and enzyme loadings were varied as stated in the text. Hydrolysis was performed at 3% solid loading, 50°C, and 200 rpm rotation, with samples collected after 72 h.

The response of glucose and xylose yields using different enzyme loadings is seen in Figure 2. In general, higher enzyme loadings led to greater sugar production, as expected. However, due to the high costs of enzymes, high enzyme loadings are unlikely to provide maximum economic benefit. As the material cost of enzymes is unknown, the economic optimal enzyme loading is currently unknown and will likely change with future research into enzyme combinations and production. Instead, the maximum sugar yields produced using at most 15 mg enzyme/kg biomass was used to determine optimal enzyme loadings. Using this constraint, optimal enzyme loadings and sugar yields are shown in Table 3.

**Table 3:** Optimal enzyme loadings for enzymatic hydrolysis of four harvests of switchgrass.

	CIR: July	CIR: October	Alamo: July	Alamo: October
Accelerase^a^	5.0	6.4	5.0	5.0
Novozyme^a^	0	0	0	0
Monomer xylanase^a^	5.0	3.6	5.0	5.0
Monomer pectinase^a^	5.0	5.0	5.0	5.0
Predicted sugars^b^	523	411	409	557
Actual sugars^c^	521	410	410	445
Sugar yield (%)	89.0%	60.9%	64.0%	68.9%
Glucose yield^c^	321	223	210	237
Xylose yield^c^	200	187	201	208
R^2^	81.3%	76.5%	81.5%	64.9%

^a^All values are in mg enzyme per g dry switchgrass; ^b^monomeric sugars released (g/kg switchgrass) after 72 h of hydrolysis as predicted by the model; ^c^actual monomeric sugars released (g/kg switchgrass) after 72 h of hydrolysis.

CIR = Cave-in-Rock.

The model gave a strong approximation of the data for three harvests, although the correlation coefficient for the October Alamo harvest was fairly low (65%). In this instance, the model did not accurately predict experimental results, overpredicting the true value. Despite this, individual enzyme conditions suggest that the optimal enzyme loading at 15 mg/g total loading is similar to the value predicted by the model. Increasing the Accelerase loading beyond 5 mg/g biomass did not greatly improve sugar yields, while including both pectinase and xylanase were necessary for high yields.

Despite different conditions providing varying amounts of cellulase and hemicellulase, there was also a reasonably strong correlation between glucose and xylose yields for all harvests studied (data not shown). This suggests a degree of synergy between glucan and xylan hydrolysis regardless of the enzyme used. Given how closely glucan and xylan polymers are intertwined with each other in the cell wall, this is not an unexpected result. Furthermore, it should be noted that each of the enzyme complexes contain activities on several different compounds [25], and so the cellulase mixtures also contain hemicellulase activity and vice versa. Increased breakdown of cellulose in cellulase-rich enzyme conditions may be increasing accessibility to the xylan, and vice versa in hemicellulase-rich enzyme conditions, thus explaining the strong correlation.

The CIR switchgrass harvested in July produced significantly more glucose than the other three harvests, yet released similar amounts of xylose. In contrast, the Alamo October switchgrass produced substantially more xylose than either the CIR October or Alamo July switchgrass, despite a similar range of glucose released. Sugar yields at or exceeding 500 g/kg biomass were seen at relatively low (<20 g/kg biomass) enzyme dosage for the Alamo October and CIR July harvests, suggesting strong potential as cellulosic feedstocks. While yields increased for the CIR October harvest compared to no additional enzymes, the overall conversion remains low. Overall sugar yield was high only in the case of CIR switchgrass harvested in July (90% total yield), while the other three yields ranged from 60% to 70%.

These values are comparable to previous research, although due to the variety of harvest locations and times as well as switchgrass ecotypes, exact comparisons cannot be made. Previous research on AFEX-treated switchgrass produced 73% sugar yield after 72 h, although the harvest date and location were unknown for this sample [18]. Dien *et al*. obtained approximately 75% sugar yield using dilute acid pretreatment on a late harvest CIR switchgrass grown in Nebraska [13], while Hu *et al*. obtained 59% to 90% yield from an unknown harvest in Virginia using alkali/microwave pretreatment [11].

Harvest date and cultivar/location do not appear to affect the composition of the enzyme complex required to break down the biomass, despite the different compositions of the biomass. With the exception of the October harvest of CIR switchgrass, all switchgrass harvests obtained optimal yields using an equal blend of Accelerase, Multifect Pectinase, and Multifect Xylanase. For the CIR October harvest, slightly more pectinase is needed than xylanase. Strong responses to the hemicellulases suggest that the AFEX pretreatment is not completely separating the cellulose from the surrounding cell wall material in any substrate. Although small amounts of cellobiose were seen in hydrolysates without Novozyme 188, its presence did not have a positive impact on sugar yields. Multifect Pectinase also contains strong b-glucosidase activity [25], likely eliminating the need for Novozyme 188. Increasing Accelerase beyond 5 g/kg produced only modest increases in sugar yields, which are unlikely to be economically competitive.

### Rate of hydrolysis

**Figure 3:**
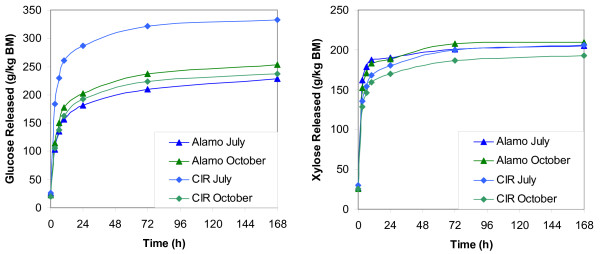
**Rate of hydrolysis**. Glucose (right) and xylose (left) released during enzymatic hydrolysis between 3 and 168 h of residence time. Pretreatments were performed at the conditions listed in Table 2 and enzyme addition as listed in Table 3. Hydrolysis was performed at 3% solid loading, 50°C, and 200 rpm rotation.

All harvests of switchgrass showed a rapid response to enzyme addition, as seen in Figure 3. As expected, both glucose and xylose released during hydrolysis rose rapidly within the first 24 h, with a slow release afterwards. Interestingly, xylose was released faster than glucose for all samples except the July harvest of CIR switchgrass. The initial rate (defined as sugar release within the first 3 h) for xylose was between 35 to 45 g/kg/h for xylose compared to 25 to 30 g/kg/h for glucose. In addition, glucose released after 168 h was 25% higher than 24 h for all harvests except for the CIR July harvest (which was 16% higher). Xylose at 168 h compared to 24 h was only 8% to 14% higher for all harvests. The xylan appears to be readily accessible to enzymes after AFEX pretreatment relative to cellulose and responds rapidly to enzymatic attack with high xylanase addition.

With the exception of the CIR July harvest, the trends for glucose released were similar in all harvests tested. These three harvests saw a similar initial rate, with the primary differences appearing between 3 to 24 h of hydrolysis. In comparison, the CIR July harvest showed a very rapid initial release of glucose, with nearly all glucose released within 24 h. It is clear that AFEX pretreatment is very effective in opening up cellulose to enzymatic attack in this harvest relative to the other three harvests, likely due to being the most immature sample. Further research is needed to determine specifically what factors influence this immediate glucose release.

The harvest location or type appears to have a greater effect on xylose released than harvest date. The Alamo harvests showed a significantly higher initial rate (42 to 45 g/kg/h) of xylose release than the CIR harvests (34 to 35 g/kg/h). In addition, a greater proportion of the total xylose release was seen within the first 24 h for the Alamo harvests compared to CIR harvests.

### Fermentation

**Figure 4:**
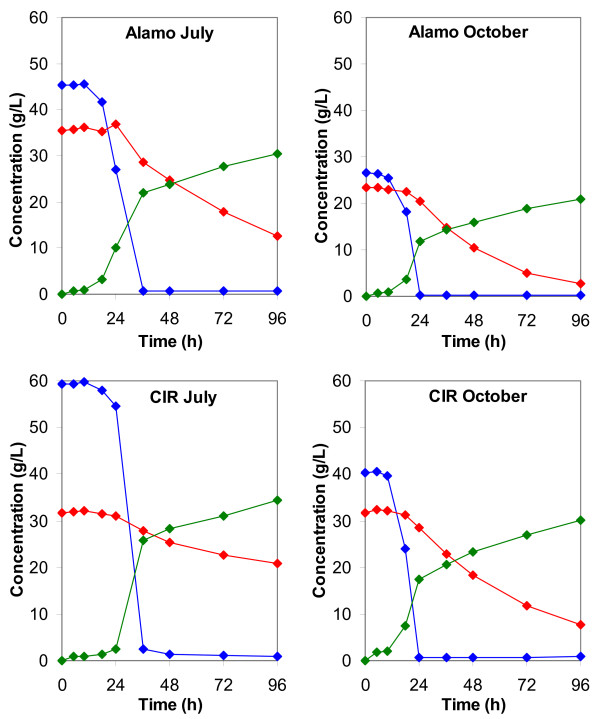
**Fermentation profiles**. Glucose (blue), xylose (red), and ethanol (green) concentrations achieved during fermentation of switchgrass. All fermentations were performed using *Saccharomyces cerevisiae *at 30°C and shaken at 150 rpm. Switchgrass hydrolysate after 72 h of enzymatic hydrolysis at 20% solid loading was used except for the October harvest of Alamo switchgrass, where the solid loading was 10%. Data points are the average of duplicate fermentations.

Glucose, xylose, and ethanol concentrations for fermentations of the four switchgrass harvests are shown in Figure 4. Metabolic yield (defined as a percentage of the theoretical amount of ethanol produced from the consumption of sugars) was high in all cases, ranging from 87% for the Alamo July harvest to 97% for the CIR July harvest. Glucose fermentation was also complete, with virtually all glucose consumed within 48 h. However, a lengthy lag phase that lasted approximately 10 to 24 h was present in all switchgrass harvests. This may be due to the strain being prepared on YEP media rather than hydrolysate, and may be eliminated if the strain were adapted to AFEX hydrolysate prior to inoculation [26]. The three hydrolysates at 20% solid loading all achieved final ethanol concentrations in excess of 30 g/l. While this value is lower than the 40 g/l threshold generally accepted for ethanol production [27], this indicates the fermentability of AFEX-treated switchgrass, and further optimisation and technology improvements will likely improve these results.

Xylose utilisation was much slower relative to glucose and was not completely consumed within 96 h. This trend has also been seen with corn stover using the same micro-organism [20]. Over two-thirds of the xylose was consumed within 96 h for three of the harvests, a satisfactory result. However, only approximately one-third of the xylose in the CIR July harvest was consumed. This is due primarily to a longer lag phase than the other harvests. After 24 h, the cell density in the CIR July harvest was 40% of the value of the Alamo July harvest and 23% of the value of CIR October. This harvest has much higher amounts of solubles present relative to the other harvests, and the ammonia may be reacting with these molecules to produce fermentation inhibitors. Further research is needed to determine why this harvest is unable to ferment as well as other harvests. Acetate levels were fairly constant throughout fermentation, ranging from 1.3 to 1.9 g/l at 20% solid loading.

### Implications

The different responses to harvest date and location/ecotype have large implications for biomass refining. Harvesting early in the season provides lower costs for pretreatment and higher potential ethanol yields, which may help offset the costs of a second harvest. With the potential additional revenue from coproducts such as proteins, the benefit for early harvests appears strong. However, any pretreatment facility will likely be designed to satisfy both harvests, and so reductions in capital costs may not actually occur. In addition, if yields from the stand decrease over time due to multiple harvests [7], a multiple harvest scenario may not be an economically viable for agronomic reasons.

Also of concern is the fact that different harvest locations or different types vary in response to pretreatment and hydrolysis. The CIR switchgrass, grown in Michigan, begins growing later in the season and senesces earlier than the Alamo switchgrass grown in Alabama. As such, while the harvest dates were similar, the relative maturities of the two varieties were different. As the CIR switchgrass was more digestible in July and less digestible in October than the Alamo material, the different relative maturities appear to have more effect on digestibility than the types of cultivars. It remains to be seen if different cultivars harvested in the same region and same season react differently to pretreatment and hydrolysis parameters. Thus, harvest practices in the northern latitudes may be adjusted to avoid the highly recalcitrant late harvest material.

## Conclusions

Fermentable sugars are effectively released from switchgrass using AFEX pretreatment and enzymatic hydrolysis. Relatively mild pretreatment results in high ethanol yields for early harvest material, while more severe conditions are necessary for later harvests. A mixture of enzymes, including xylanase and pectinase, are required to release the greatest amounts of sugar. Xylan was digested faster than glucan for all types of biomass, with an initial rate of 35 to 45 g/kg/h xylose released compared to 25 to 30 g/kg/h glucose. The upland CIR switchgrass from Michigan harvested in July showed the greatest response to pretreatment and hydrolysis, producing 520 g monomeric sugar per kg biomass, while harvesting in October showed the least response, producing 410 g/kg biomass. All hydrolysates were fermentable, although the July CIR harvested material showed poor xylose utilisation.

Harvest date and location/ecotype have a substantial impact on AFEX pretreatment conditions and sugar released, although not a major factor in enzyme requirements. For the northern CIR switchgrass used in this study, large differences were seen in pretreatment conditions and response between the two harvests, while this difference was muted in the southern Alamo switchgrass. Although this suggests that the harvest location is the dominant response, as the northern climate has a shorter growing season than the southern climate, further research is required to separate the effects of harvest location and ecotype. Thus, harvesting strategies must be tailored to local needs in order to maximise ethanol production. An integrated approach, considering long-term viability of stands, biomass yield on the farm, pretreatment processing conditions and cost, and resulting ethanol yields simultaneously, is necessary to determine the optimal solution in satisfying both farmers' and ethanol producers' needs. In addition, these factors would affect the possibility of producing coproducts switchgrass along with bioenergy.

## Competing interests

The authors declare that they have no competing interests.

## Authors' contributions

BB designed the hydrolysis experiments, performed the statistical analysis, and drafted the manuscript. CR carried out the enzymatic hydrolysis experiments. MJ designed and carried out the fermentation experiment. VB participated in the design of the study and analysis. BD participated in the design and coordination of the study. All authors provided critical input to the manuscript, and read and approved the final manuscript.
